# Supplementary material for: MetaRibo-Seq measures translation in microbiomes
Source: Nat Commun. 2020 Jun 29;11:3268. doi: 10.1038/s41467-020-17081-z (PMC7324362; doi:10.1038/s41467-020-17081-z)
Supplement: Supplementary file 10 — Supplementary Data 7 [file 41467_2020_17081_MOESM10_ESM.zip › File2/Confidence_High_Taxonomy/223837_out.krona.html]

Javascript must be enabled to view this page.

members
magnitude
magnitudeUnassigned
count
unassigned
taxon
rank

223837\_out

6

superkingdom
2
6

6
1239
phylum

186801
class
6

186802
order
6

6
family
541000

6
216851
genus


SRS014948\_contig\_number\_1151SRS019496\_contig\_number\_14693SRS050422\_contig\_number\_36617SRS063518\_contig\_number\_33409SRS144135\_contig\_number\_34057SRS146812\_contig\_number\_33655
6
species
853
